# The Expanded Natural History of Song Discography, A Global Corpus of Vocal Music

**DOI:** 10.1162/opmi.a.4

**Published:** 2025-07-07

**Authors:** Mila Bertolo, Martynas Snarskis, Thanos Kyritsis, Lidya Yurdum, Constance M. Bainbridge, S. Atwood, Courtney B. Hilton, Anya Keomurjian, Judy S. Lee, Alex Mackiel, Vanessa Mak, Mijoo Shin, Alma Bitran, Dor Shilton, Lana Delasanta, Hang (Heather) Do, Jenna Lang, Tenaaz Irani, Jayanthiny Kangatharan, Kevin Lafleur, Nashua Malko, Quentin D. Atkinson, Manvir Singh, Samuel A. Mehr

**Affiliations:** Integrated Program in Neuroscience, McGill University, Montreal, QC, Canada; Centre for Research on Brain, Language, and Music, McGill University, Montreal, QC, Canada; International Laboratory for Brain, Music, and Sound Research, Department of Psychology, University of Montreal, Montreal, QC, Canada; Child Study Center, Yale University, New Haven, CT, USA; School of Psychology, University of Auckland, Auckland, New Zealand; Department of Psychology, University of Amsterdam, Amsterdam, The Netherlands; Department of Communication, University of California, Los Angeles, Los Angeles, CA, USA; Department of Psychology, Princeton University, Princeton, NJ, USA; Department of Psychology, Harvard University, Cambridge, MA, USA; Department of Psychology, University of Chicago, Chicago, IL, USA; Department of Psychology, University of British Columbia, Vancouver, BC, Canada; Department of Government, Harvard University, Cambridge, MA, USA; Department of Clinical Psychology, Rutgers, The State University of New Jersey, Newark, NJ, USA; Cohn Institute for the History and Philosophy of Science and Ideas, Tel Aviv University, Ramat Aviv, Israel; Sidney M. Edelstein Center for History and Philosophy of Science, Technology and Medicine, Hebrew University, Jerusalem, Israel; Department of Psychological Sciences, University of Connecticut, Storrs, CT, USA; Center for the Ecological Study of Perception & Action, University of Connecticut, Storrs, CT, USA; University of Pennsylvania Graduate School of Education, Philadelphia, PA, USA; Faculty of Medicine, University of Ottawa, Ottawa, ON, Canada; Max Planck Institute for the Science of Human History, Jena, Germany; Department of Anthropology, University of California, Davis, Davis, CA, USA

**Keywords:** music, corpus, cross-cultural, song

## Abstract

A comprehensive cognitive science requires broad sampling of human behavior to justify general inferences about the mind. For example, the field of psycholinguistics relies on a rich history of comparative study, with many available resources that systematically document many languages. Surprisingly, despite a longstanding interest in questions of universality and diversity, the psychology of music has few such resources. Here, we report the *Expanded Natural History of Song Discography*, an open-access corpus of vocal music (*n* = 1007 song excerpts), with accompanying metadata detailing each song’s region of origin, language (of 413 languages represented here), and one of 10 behavioral contexts (e.g., work, storytelling, mourning, lullaby, dance). The corpus is designed to sample both broadly, with a large cross-section of societies and languages; and deeply, with many songs representing three well-studied language families (Atlantic-Congo, Austronesian, and Indo-European). This design facilitates direct comparison of musical and vocal features across cultures, principled approaches to sampling stimuli for experiments, and evaluation of models of the cultural evolution of song. In this paper we describe the corpus and provide two proofs of concept, demonstrating its utility. We report (1) a conceptual replication of previous findings that the acoustical forms of songs are predictive of their behavioral contexts, including in previously unstudied contexts (e.g., children’s play songs); and (2) similarities in acoustic content of songs across cultures are predictable, in part, by the relatedness of those cultures.

## INTRODUCTION

Investigating music across contexts and cultures is essential for understanding the interacting biological and cultural underpinnings of the human capacity for music. Findings of universality suggest what characteristics of musicality are likely to reflect basic aspects of the human music phenotype. For example, the mutual intelligibility of music across cultures (Hilton et al., 2023; Singh & Mehr, 2023; Yurdum et al., 2023), early psychophysiological responses to culturally unfamiliar music in infancy (Bainbridge et al., 2021), and cross-culturally common usage of certain rhythms (Jacoby et al., 2024) support predictions of some adaptive functions of musicality in the domain of communication (e.g., Mehr et al., 2021; Trehub, 2001). Findings of diversity suggest what characteristics of musicality are molded by cultural forces and in what ways. For instance, cross-cultural variability in preferences for consonance over dissonance (McDermott et al., 2016) suggest mechanisms of cultural evolution may shape musical aesthetics. Moreover, differences in the propensity to match pitch across octaves (Jacoby et al., 2019) and effects of tonal language experience on music processing abilities (Liu et al., 2023) suggest that cultural experience can mold lower-level music perception—despite key aspects of music perception showing evidence for universality (Mehr, 2025).

While research in the psychology of music has had a recent increase in focus on cross-cultural studies, like many of the cognitive sciences the bulk of music research is still carried out in Western, Educated, Industrialized, Rich, and Democratic (WEIRD) societies, using stimuli from these same societies. This, of course, paints a non-representative picture of musicality (Jacoby et al., 2020). One route forward is the diversification and expansion in scope of global music corpora (Savage, 2022).

This is not a new idea: collecting large numbers of samples from diverse participants is common in many fields. Psycholinguists often unite corpus work with empirical studies, improving the ecological validity of their work (Gilquin & Gries, 2009). For example, analysis of a naturalistic corpus of spoken conversation showed that turn-taking happens more than twice as rapidly as previously shown in laboratory studies (Reece et al., 2023). In the field of emotion perception, a study that used an expansive set of stimuli (over 2000 recordings of vocal bursts from people in four countries) mapped 24 distinct clusters of emotion categories and their overlap (Cowen et al., 2019). A related approach to animal vocalizations revealed no apparent relation between the magnitude of a species’ sexual dimorphism in size and the pitch or complexity of birdsong, contrary to what would be predicted by sexual selection accounts of birdsong evolution (Pearse et al., 2018). The use of large, diverse, and naturalistic corpora can also mitigate the over-representation of English in cognitive science research (Blasi et al., 2022).

What of music? While the availability of corpora in the music domain has increased, most focus on popular or classical music, (e.g., the *Million Song Database*, Bertin-Mahieux et al., 2011; *MusicBrainz*, Swartz, 2002; the *Google/Magenta MAESTRO dataset of piano performances*, Hawthorne et al., 2018). Only a few attempt global representation of music (reviewed in Panteli et al., 2017; Savage, 2022), such as *The Garland Encyclopedia of World Music* (Nettl et al., 2002), the *Natural History of Song Discography* (Mehr et al., 2019), *The Global Jukebox* (Wood et al., 2022), and the *Compmusic project* (Serra, 2014). A few global corpora also focus on narrower contexts; these include infant-directed vocalizations (Hilton et al., 2022), lullabies (Trehub et al., 1993a), and a group of researchers producing speech and song themselves (Ozaki et al., 2024).

These music corpora vary in the availability of audio for each musical excerpt; the presence or absence of symbolic or data-analytic representations of the music; and the depth of available metadata, both for musical excerpts themselves (such as the behavioral context of a song) and the people producing the music (such as the language or dialect of the lyrics).

Here, we report the *Expanded Natural History of Song Discography*, a corpus of 1007 excerpts of vocal music designed to support research on both the universality and diversity of human song (following Mehr et al., 2019). The sampling strategy includes both broad global representation and deep sampling of three large language families (Austronesian, Atlantic-Congo, and Indo-European). Given the well-known relevance of behavioral context to the forms of vocal music (e.g., Yurdum et al., 2023), the corpus samples songs across 10 behavioral contexts (Table 1) with clearly defined inclusion and exclusion criteria. To maximize usability, we used standardized descriptors of all songs’ metadata: behavioral context, region of origin, and the language in which the song was produced.

**Table 1.** Definitions of Behavioral Contexts. Each of the 10 behavioral contexts were defined by inclusion and exclusion criteria.

Behavioral Context	Inclusion Criteria	Exclusion Criteria
Dance	Sung with the goal of a person or persons dancing along to it.	Songs that happen to be accompanied by dancing but are used for other goals.
Healing	Sung in a healing ceremony with the goal of curing sickness.	Songs describing sick people or a past epidemic.
Love	Sung to express love directly to another person or to describe currently felt love.	Songs about unrequited love, deceased loved ones, or love for animals or property.
Lullaby	Sung to an infant or child with the goal of soothing, calming, or putting to sleep.	Songs designed to excite the listener (e.g., play songs); singing games.
Play	Sung to excite a child or infant and engages them in play. This can include singing games.	Children’s songs for soothing, calming, or putting to sleep.
Procession	Sung to accompany a formalized march, entrance, or parade, such as during a wedding, funeral or the introduction of a leader.	Processions of dancing.
Mourning	Sung to express grief or sadness about the death of a person, in the present or past.	Songs for sick or dying people, or laments about events other than the death of a person.
Work	Sung to accompany work activities, including planting, grinding, harvesting, processing, tool-making.	Hunting songs (to celebrate successful hunts, prepare for hunts).
Story	Sung to recount historical or mythological events, narrate a sequence of activities by one or more persons.	Lullabies that include stories.
Praise	Sung to express admiration for the traits or accomplishments of a person, animal, location, or item of property.	A song expressing love for another person or explicitly religious songs (like devotionals).

Finally, and most importantly, the corpus, including all audio excerpts and metadata, is open-access, with no restrictions on its noncommercial use (readers may access it on Zenodo at https://doi.org/10.5281/zenodo.8223168).

## CORPUS CONSTRUCTION

We built the corpus in five stages.

**Figure 1.**
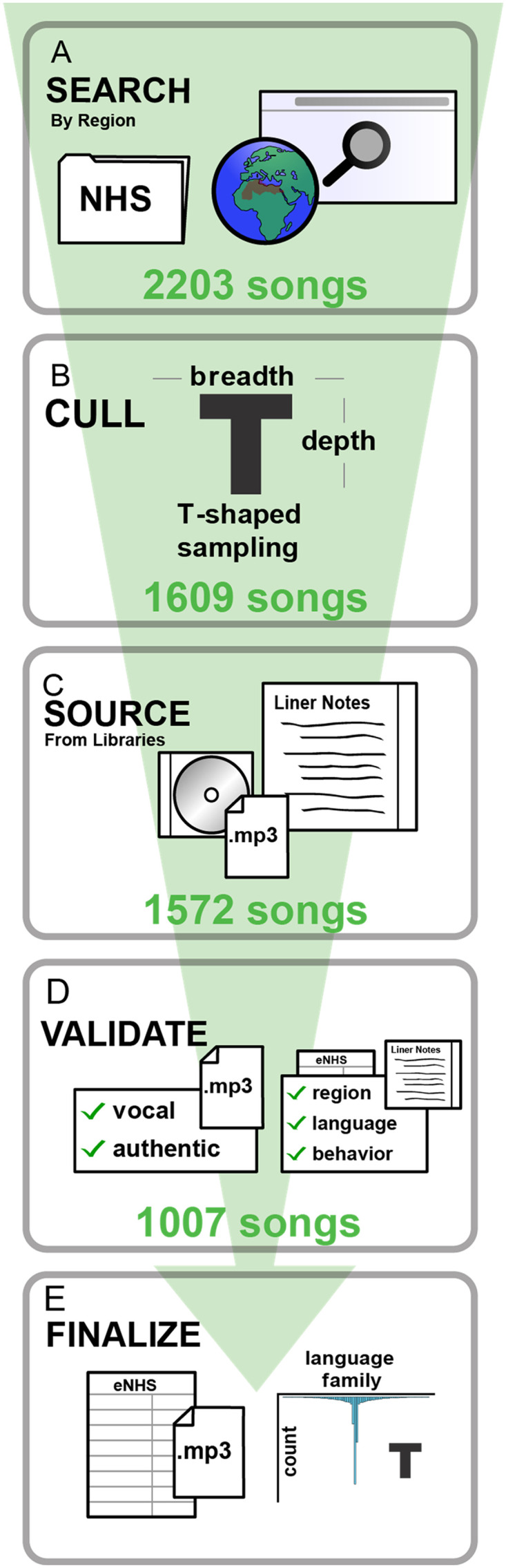
**How we built the corpus**. (A) We gathered candidate items from libraries and online databases via broad initial searches. (B) To create a “deep” sample that densely sampled from three large language families for closer study, we retained all items from the Atlantic-Congo, Austronesian, and Indo-European language families. To create a “broad” sample that was diverse and did not over-represent any particular language family, we culled the initial search by selecting a maximum of 2 songs per language family per behavioral context (in families other than Atlantic-Congo, Austronesian, and Indo-European). (C) Audio recordings of candidate items were sourced digitially or by digitizing from a physical CD or LP, and associated liner notes were scanned. (D) Two independent groups of annotators validated the metadata for each recording; each group reviewed the liner notes for each recording and determined whether there was sufficient information therein to determine the recording’s region, language, and behavioral context. When two annotators disagreed on such determinations, a third annotator arbitrated, or a consensus decision was reached after discussion. (E) The corpus was released on Zenodo, in the form of a CSV metadata file and with each audio recording excerpted as a 10-second MP3 file; metadata was also provided to the D-PLACE database.

First, research assistants searched for candidate items (Figure 1A). They were each assigned a geographical region and instructed to seek out all available recordings of songs used in each of ten behavioral contexts (Table 1). They did so using a variety of public and private sources, including internally via the Harvard Libraries (e.g., the Archive of World Music at Loeb Music Library); at various other libraries and institutes (e.g., the Centre de Recherche en Ethnomusicologie at Université Paris Nanterre, the British Library, etc.); via library aggregation databases (e.g., WorldCat); recordings previously used in the original *Natural History of Song Discography* (Mehr et al., 2019); and by directly contacting ethnomusicologists, music collectors, and scholars working with private collections of field recordings. Recordings were only considered to be positive candidates for inclusion if they were accompanied by credible information concerning (i) the recording’s geographical region of origin; (ii) the language the recording was sung in; and (iii) source ethnography supporting the behavioral context of the recording. While these types of information were typically included in liner notes to a published album or a library-based field recording, in some cases they were provided in referenced publications (e.g., a book that accompanied a recording), or were provided informally (e.g., via e-mail from a collector). This process yielded 2203 candidate items.

Second, we culled the candidate items to an initial sample (Figure 1B), whose structure balanced *broad* sampling with large, global diversity, and *deep* sampling in select areas for more detailed study. To maximize global diversity (for the *broad* portion of the sample), we retained at least one candidate item for each behavioral context type for each additional language family and major language. For each of the 10 song behavioral contexts, if any language family (other than Indo-European, Austronesian, and Atlantic-Congo) had more than 2 songs, we randomly selected only 2 of them in an effort to avoid over-representing any particular language family. To maximize within-language-family representation in the *deep* sample, we retained all songs available representing Indo-European, Austronesian, and Atlantic-Congo languages. This process yielded 1609 items.

Third, we obtained recordings from their original sources (e.g., using Interlibrary Loan, purchasing items, requesting digital audio files from collectors) and extracted an audio excerpt from each recording. To comply with Fair Use (under the United States doctrine), we extracted a brief excerpt of each recording, as in prior work (Mehr et al., 2019); we did this by using a random number generator to indicate a starting timestamp within the song and cutting the following 10 seconds of audio. If the excerpt did not contain audible singing (e.g., if it only contained instrumental music), a new random timestamp was generated until this condition was met. This process yielded 1572 song excerpts.

Fourth, two teams of two coders independently reviewed each excerpt (Figure 1D). They confirmed (i) that the excerpt contained audible singing; and (ii) did not appear to be an unfaithful recreation of an original song, such as an electronic or orchestral rendition of a traditional song, or a studio-based performance of a song that was obviously unrelated to its original context. Then, they conducted a detailed review of the source ethnography (e.g., liner notes) to ensure that the excerpt’s metadata (i.e., language classification, behavioral context, and geographic region) were accurate. Each of the two teams carried out their reviews without access to the other team’s review. Another researcher then compared the two teams’ reviews; when assessments did not agree, they arbitrated between the two judgements, reviewing all original source materials, and deciding whether to remove a recording from the sample. The researchers’ reviews thus occasionally overrode initial assessments of each song’s metadata (region of origin, language, behavioral context), and resulted in a small number of language families outside the 3 large language families having more than 2 songs per behavioral context. This process yielded the complete corpus of 1007 song excerpts.

Finally, we prepared the corpus for public release (Figure 1E). We used Adobe Audition to normalize the loudness and add 1-second fades at the beginning and end of each excerpt, to facilitate their use in experiments. We released the corpus publicly on Zenodo (https://doi.org/10.5281/zenodo.8223168), including all audio files and a metadata table. Spreadsheets detailing each stage of song search, song sourcing, and validation are in this paper’s GitHub repository (https://github.com/themusiclab/nhs-expanded).

We also provided a version of the corpus to *D-PLACE: The Database of Places, Language, Culture, and Environment* (Kirby et al., 2016), which stores the *Expanded Natural History of Song Discography* in a dedicated Github repository (https://github.com/D-PLACE/dplace-dataset-ccmc) in Cross-Linguistic Data Format (Forkel et al., 2018), an approach that facilitates analyzing the corpus alongside other cross-cultural and cross-linguistic datasets. As in other iteratively-updated corpora concerning human behavior (e.g., *Saraga*, a dataset of Indian art music, Srinivasamurthy et al., 2021), this format allows users to suggest changes to the corpus by submitting pull requests, which the managers of the repository can review and approve.

### Annotation Details

We annotated each song with three pieces of metadata: (1) *region*, the geographic region in which it was recorded; (2) *glottocode*, the languoid (i.e., a language or dialect) in which it was sung; and (3) *type*, the behavioral context surrounding its original use.

We classified geographic regions with a predetermined set of world regions defined by the *Electronic Human Relations Area Files* (eHRAF) (Human Relations Area Files, 1967)^1^, following our previous work (Mehr et al., 2019). In addition to the original 35 eHRAF regions we included Western Europe, Central Europe, and Eastern Europe, which at the time of writing are not included in eHRAF.

We classified languoids via the *Glottolog* database (Hammarström et al., 2023), using as precise a languoid level as the available information allowed. For example, for some songs, the supporting liner notes were sufficiently detailed that annotators, unaware of each other’s work, identified the same specific dialect that a song was sung in. In others, annotators could only reach consensus on the broader language. The use of glottocodes allows each song to be linked to a persistent identifier with a known location in the genealogy of all the world’s languages, and allows this corpus to be integrated with other existing cross-cultural datasets that use such identifiers, such as *D-PLACE* (Kirby et al., 2016), *NoRaRE* (Tjuka, 2021), *PHOIBLE* (Moran & McCloy, 2019), *Lexibank* (List et al., 2022), and *Grambank* (Skirgård et al., 2023). Glottocodes are often used in phylogenetic analyses, for example, to study questions surrounding the evolution of syntactic structure (Hahn & Xu, 2022). They can similarly be used to study the evolution of musical forms.

To determine song behavioral context, research assistants read the songs’ associated liner notes to confirm whether each song fit an inclusion criterion for any of the behavioral contexts we considered here, and did not violate its exclusion criteria (Table 1). The present corpus considers ten behavioral contexts; the four considered in the original *Natural History of Song Discography* (i.e., dance, healing, love, lullaby), along with six newly added behavioral contexts: play, mourning, work, praise, story, and procession; see Table 1. These six were chosen by surveying the existing literature for candidate song contexts that appeared across distant human societies. Some songs plausibly aligned with more than one behavioral context definition (see Discussion); in these cases, research assistants chose what they determined the liner notes communicated about the song’s *primary* function, even if the song could plausibly be represented by more than one context.

For both geographic region and glottocode, when the supporting evidence was ambiguous, annotators made judgments by consensus. For example, in NHS2-U13X, the liner notes describe the general cultural context being the music of the Minnesota Ojibway, suggesting the eHRAF region *Plains and Plateau*, but also describe exchange with Ojibway who live over the Canadian border, where songs were actually recorded; annotators ultimately chose the eHRAF region *Arctic and Subarctic* for this song. In cases where glottocode was ambiguous, annotators could reach consensus by using a more general glottocode at a higher level of the language tree. For example, in NHS2-2BT6, where liner notes state this recording is from Tiga Island, which currently has no corresponding glottocode; instead, annotators ultimately chose a higher level of linguistic granularity, with the *Loyalty Islands* languoid. Shifts between these levels of granularity make it difficult to measure how many distinct societies the corpus actually represents; the 413 languoids labelled in the corpus likely reflect a lower bound on the number of societies represented.

### Breadth and Depth of the Corpus

**Figure 2.**
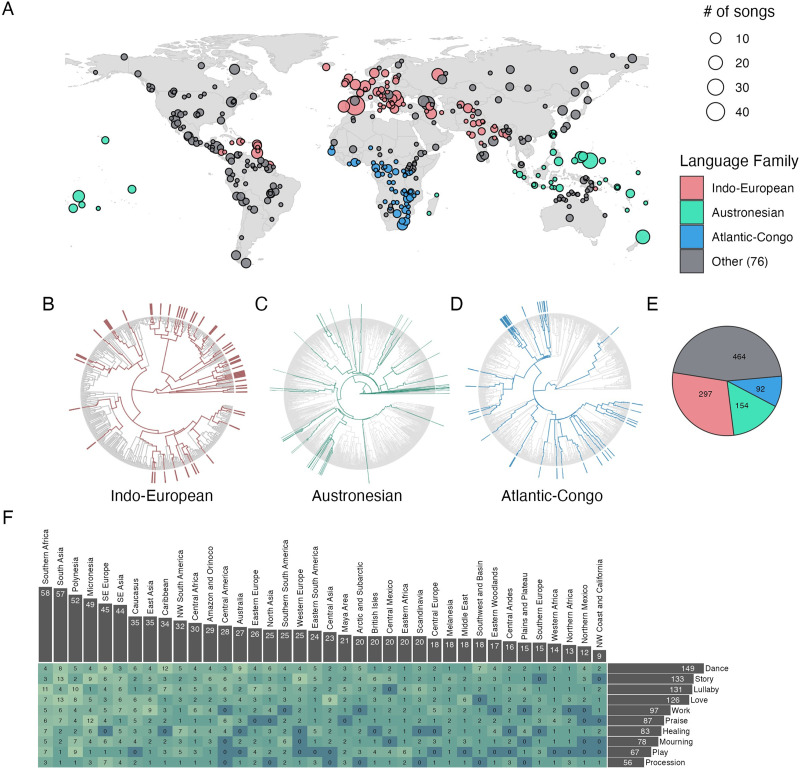
**The Expanded Natural History of Song Discography**. (A) The 1007 songs in the corpus are globally distributed. The size of each bubble represents the number of songs in the corpus from the approximate map location. Locations are plotted using Glottolog metadata and aggregated by glottocode (i.e., languoid); 47 locations, representing 88 songs, are omitted due to missing location data. (B–E) The linguistic distribution is broad, with approximately half the corpus representing three large language families, deeply sampled; and the other half from a large number of other language families (see the pie chart in E). In the phylogenetic language trees, lineages are highlighted in color when represented by at least one song. (F) The distribution of behavioral contexts represented in the corpus is also broad; the heatmap depicts the number of songs representing each geographic region in each of the ten behavioral contexts studied. Of the 380 possible region-by-behavioral-context pairings, 328 are represented by at least one song.

The *Expanded Natural History of Song Discography* contains 1007 songs, with broad global and linguistic distributions (Figure 2A–E). The sampling strategy yielded a broad diversity of songs across languages (*n* = 413 languoids, median songs per languoid = 1, range 1–46); behavioral contexts (10 types, median songs per type = 92, range 56–149); and geographic regions (median songs per region = 23, range 1–58). Of the 380 possible behavior-by-region combinations, 329 are represented by at least one song (Figure 2F).

The strategy to deeply sample songs across three language families (Austronesian, Indo-European, and Atlantic-Congo) yielded many songs (543 of the 1007 songs) with languages hailing from diverse branches within each language family (Figure 2B–D). This mixed approach to sampling (both a deep and broad sample) should facilitate phylogenetic analyses, while still maintaining a large representation of language families corpus-wide (*n* = 79 language families).

## RESULTS

We undertook two sets of analyses. The first focussed on the breadth of the corpus, to test a question of universality; and the second focussed on the depth of the corpus, to test a question of diversity.

### Proof-of-Concept 1: Acoustic Forms Predict the Behavioral Contexts of Songs

We and many others have argued that aspects of music are mutually intelligible across cultures (Argstatter, 2016; Balkwill & Thompson, 1999; Balkwill et al., 2004; Fritz et al., 2009; Hilton et al., 2023; Laukka et al., 2013; Mehr et al., 2018, 2019; Sievers et al., 2013; Trehub et al., 1993b; Yurdum et al., 2023; see Singh & Mehr, 2023 for a review). A pattern of mutual intelligibility implies links between form and function, akin to those found in vocalizations found in non-human primates (Filippi et al., 2017; Owren & Rendall, 2001); frogs (Wagner, 1989); hawks (Mueller, 1971); and deer (Clutton-Brock & Albon, 1979). In music, form-function links are predicted by functional accounts of musical behavior (e.g., Kotler et al., 2019; Mehr & Krasnow, 2017; Mehr et al., 2017, 2021).

While studies of form-function links in music include a large diversity of *listeners*, they use a limited diversity of music as stimuli. For example, previous work showed an ability to detect the behavioral context of lullabies, dance songs, and healing songs in English-speaking adults recruited on Amazon Mechanical Turk (Mehr et al., 2018); in English-speaking adult citizen-science volunteers (Mehr et al., 2019); in English-speaking children (Hilton et al., 2023); and in adults recruited globally, in many languages, who were living in both industrialized and non-industrialized societies (Yurdum et al., 2023). But in all four studies, the same relatively small collection of 118 songs was studied (i.e., the original *Natural History of Song Discography*). While this collection represented an advance in the diversity of music studied in this type of experiment (Fitch & Popescu, 2019), its small size constrains the generality of findings using it.

As such, for a first proof-of-concept of the utility of the *Expanded Natural History of Song Discography*, we aimed to reproduce the findings of these previous studies, testing whether the acoustic features of songs in the corpus were reliably predictive of their behavioral functions across the cultures represented in a second, larger corpus.

We extracted statistical summaries of the acoustic content of each song excerpt using MIRtoolbox (Lartillot et al., 2008), a MATLAB toolbox for analysis of spectral and rhythmic features. We used the mirfeatures function to extract 38 acoustic features per song. The song excerpts were loudness-matched before model fitting. This tool has previously been used to quantify musical features in stimuli that elicit groove in music (Matthews et al., 2020); to describe global patterns in the acoustic features of speech and song (Albouy et al., 2024; Hilton et al., 2022); and to relate acoustical forms of songs to their behavioral contexts (Mehr et al., 2019).

We ran a least absolute shrinkage and selection operator (LASSO) model (Friedman et al., 2010) to predict the behavioral context from each excerpt’s acoustic features. To minimize bias due to differing base rates of behavioral types, including cases in which the LASSO model limits its predictions to a small subsample of the most prevalent behavioral types, we used an ensemble modelling approach in which many LASSO models were ran and their predictions were aggregated. We first randomly split the data into a 10-part test-train partition. To create predictions for each testing partition while minimizing base-rate biases, we generated new training sets by bootstrapping (i.e., sampling with replacement) from the remaining training partitions while keeping the number of behavioral types equivalent (e.g., in each partition, sampling the same number of dance songs as story songs).

For each testing partition, we bootstrapped 10 training sets and trained a LASSO model on each set, recording its predictions. We repeated this procedure for 25 randomly selected test-train splits. Finally, the results were aggregated, with the final prediction for each song recorded as the modal prediction from the 250 LASSO models. We calculated significance by performing a permutation test on the output of the model; *p*-values for the model as whole as well as each behavioural context were estimated as the proportion of permutation simulations which resulted in accuracy higher than the ensemble model.

We first used this approach to classify only songs from the four behavioral contexts represented in the original *Natural History of Song Discography* (i.e., dance, lullaby, healing, and love), providing a direct replication of prior results (see Figure 5B in Mehr et al., 2019).

**Figure 3.**
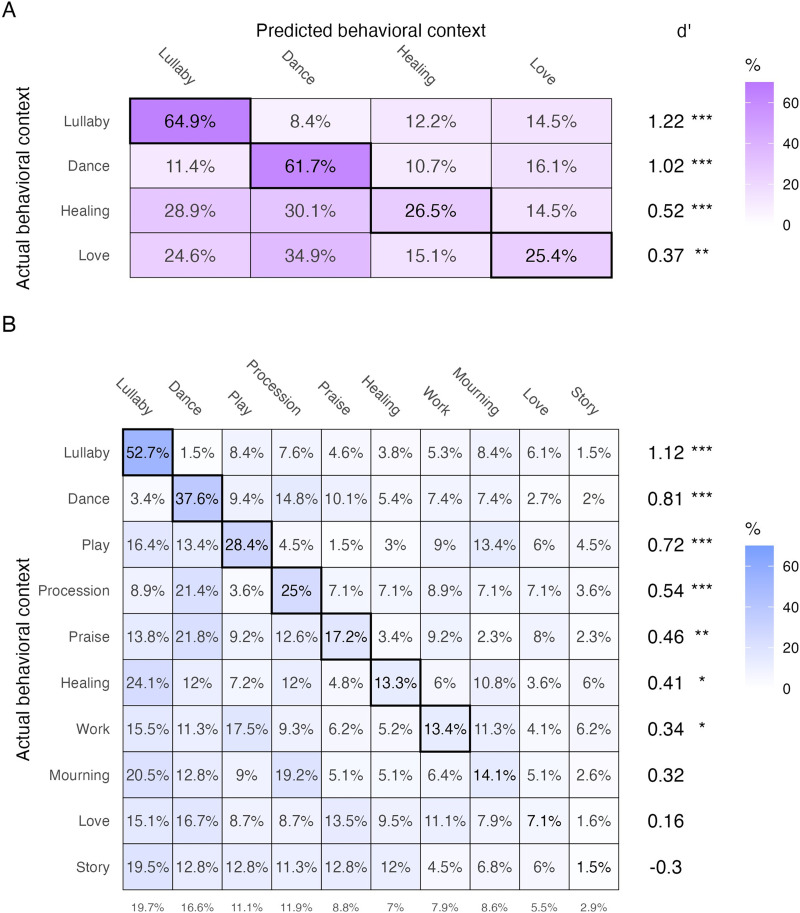
**The acoustic forms of songs predict their behavioral contexts with varying accuracy**. We trained an ensemble LASSO model to predict the behavioral context of songs using acoustic features extracted from their recordings. Significance testing was performed using a permutation test on the results of the ensemble model, with the *p*-value set to the proportion of random permutations with accuracy greater than the ensemble model for each song function (*p < .05, **p < .01, ***p < .005). The confusion matrices show (A) a conceptual replication of prior work (Mehr et al., 2019), using only the four behavioral contexts studied therein. Lullabies, dance, healing, and love songs are all predicted with significant accuracy, but sensitivity to lullabies and dance songs is far higher than for healing and love songs. This analysis approach is extended in (B) to all ten behavioral contexts studied in the *Expanded Natural History of Song Discography*.

The replication was successful (Figure 3A): the model’s classification of song behavioral contexts based on acoustic features was above chance (mean accuracy = 47.2%, chance level: 25%; *p* < .005, permutation test) and higher than in Mehr et al. (2019), where mean accuracy using MIRtoolbox features was on par with human listeners, at approximately 42% correct. Moreover, accuracy varied across song types in an identical fashion to the pattern found in the smaller corpus, where dance songs and lullabies are classified with highest accuracy, while healing songs and love songs are classified with lower accuracy (dance: *p* < .005, lullaby: *p* < .005, healing: *p* < .005, love: *p* < .01, permutation tests). Note that we define chance accuracy as the inverse of the number of behavioral contexts, rather than the proportion of songs of a given behavioural context actually present in the corpus.

Second, we expanded the approach to test whether the same acoustic features could be used to identify all *ten* behavioral contexts represented in the new corpus—six of which have not previously been studied in this fashion (i.e., play, procession, praise, work, mourning, story). We used the same LASSO ensemble modeling approach.

Here too the acoustical forms of songs were predictive of their behavioral contexts (Figure 3B). On average, performance was above chance (accuracy = 21.7%, chance level of 10%; *p* < .005, permutation test), although with ten categories of songs, variability was high (dance: *p* < .005, lullaby: *p* < .005, play: *p* < .005, procession: *p* < .005, praise: *p* < .01, healing: *p* < .05, work: *p* < .05, mourning: *p* = 0.07, love: *p* = 0.25, story: *p* = 0.92, permutation tests). Some song contexts showed very clear relations: lullabies, dance songs, and play songs all have relatively high *d*-prime scores. However, the model has no sensitivity at all to story songs, and only weakly detects love songs, conceptually replicating prior work on human classification of love songs (Yurdum et al., 2023).

These accuracy scores can be considered a lower bound for the estimate of the true dissociability between behavioral contexts, given both the large number of categories to classify and the relatively coarse, machine- extracted low-level acoustic features used by the model. Future work may help to determine whether human listeners can categorize songs representing any of the six behavioral contexts represented here that have not previously been used in experiments. If they can, and with higher accuracy than the LASSO classification reported here, it would suggest that the MIRtoolbox data captures only a subset of the acoustic differences between songs.

### Proof-of-Concept 2: Cultural Relatedness and Behavioral Context Both Explain Acoustical Similarities in Songs

Many studies explore how music perception and production are patterned across cultures, as such data are informative for hypotheses concerning the biological and cultural evolution of music (for discussion, see Mehr et al., 2021). Indeed, a variety of sources of evidence support the idea that cultural experience shapes musicality: infants show preferences for culturally familiar metrical structures over unfamiliar ones (Soley & Hannon, 2010); music processing abilities (i.e., pitch discrimination and beat alignment) differ as a function of linguistic experience (Liu et al., 2023); melodies sung in tonal languages are shaped in part by the contours of those languages (Kirby & Ladd, 2016); and so on.

Computational approaches that directly model cultural relatedness have tested a variety of hypotheses about the structure of cross-cultural diversity in musicality, complementing these experimental studies. Such approaches have revealed how frequent specific structural forms are globally, such as the near-universal use of discrete pitches and non-equidistant scales versus the large cultural variance in the use of pentatonic scales (Savage et al., 2015). And in contrast to language, which by necessity requires low within-population variability to enable communication, musical diversity throughout Austronesian languages shows more variability within languages than between them (Rzeszutek et al., 2012).

One outstanding question in the modelling of musical diversity is how the interplay between cultural diversity and the functional uses of music lead to reliable variability in musical forms. This question has been difficult to address, however, because previous corpus studies have not accounted for the behavioral contexts of the music being performed. This omission complicates the interpretation of cross-cultural models, as the music available for a given language or language family may be strongly biased. For example, if a sample of Hindi songs includes only dance music and no lullabies, model estimates for the influence of Hindi culture on musical features will be biased toward those features characterizing dance songs, even if such a shift is independent of linguistic influences.

The availability of behavioral context information for each song in the *Expanded Natural History of Song Discography* allows for a test of the relative impact of contextual factors (e.g., when a song is used for soothing a baby) on musical features, relative to the impacts of geographical location, language, and linguistic ancestry of that ethnolinguistic group on how a song sounds.

We demonstrate this approach with an example acoustic feature *spectral entropy*, which loosely tracks musical complexity. For example, spectral entropy correlates with perceived musical disorganization and phrase termination (Danieli & Frank, 2022), while melodic and textural entropy represent components of complexity relating to musical pleasure (Margulis & Beatty, 2008). Several theories predict that lullabies and dance songs should differ substantially on this feature, as lullabies tend to be identifiable in being “soft” and “simple” (Trehub et al., 1993b) and low in roughness (Hilton et al., 2022).

**Figure 4.**
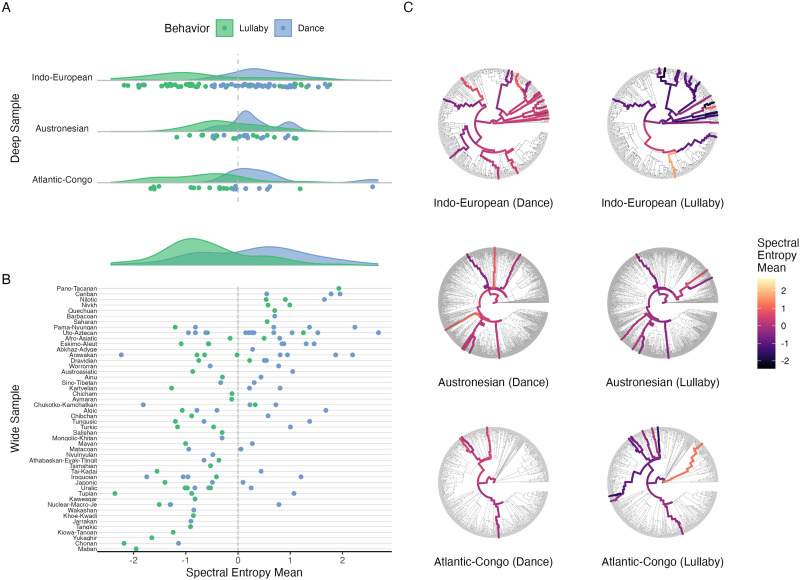
**Visual exploration of spectral entropy across cultures**. Spectral entropy, an acoustic feature that loosely tracks musical complexity, shows distinctive patterns within and between language families. (A) In each of three language families, there is a robust difference in spectral entropy between dance songs (in blue) and lullabies (in green). (B) This effect replicates across the rest of the 49 language families in which both dance songs and lullabies are represented in the corpus. (C) A within-language-family view of the same data shows variability in spectral entropy in both dance songs and lullabies within each deeply-sampled language family tree. Points at the outer edge of each circle represent individual languages, with branches inwards joining those languages to their shared linguistic clades. The branches’ colors indicate average spectral entropy for the song type indicated in the label below each dendrogram. Nodes closer to the center of the tree are colored to show the average spectral entropy of the songs included in that clade. The Indo-European lullabies, for instance, have low spectral entropy overall, with a small number of atypically high examples.

In Figure 4, we examine this feature within the three deeply-sampled language families, and across the whole corpus. The overall effect of behavioral context is evident: dance songs, as a class, are higher in spectral entropy than lullabies (Figure 4A–B). Additionally, spectral entropy values also differ between dance songs and lullabies *within each language family* (Figure 4C).

These exploratory, descriptive visualizations help reveal how acoustics vary between cultures and behavioral contexts, highlighting the substantial variability within each language family, while demonstrating the general trend that spectral entropy can coarsely differentiate between dance songs and lullabies. This association may track with differences in the purpose of dance music and lullabies, especially in terms of up- or down-regulating arousal.

We continued by asking the more general question of what the relative influences of behavioral context and phylogeny are across all the measured acoustic features (not only spectral entropy). We used Bayesian phylogenetic modeling to estimate the group-level effects on acoustic features of (a) songs’ behavioral context; (b) culture (indexed by glottocode); (c) phylogeny (i.e., linguistic ancestry); and (d) geographical proximity. The models were estimated in brms (Bürkner, 2021), modeling random group effects using Gaussian processes and computing intra-class correlation (ICC) as the proportion of variance explained by each variable. We consider the relative values of the ICCs across variables and model specifications, described below, but avoid interpreting absolute ICC values in isolation. The acoustic features used here are the same MIRtoolbox features described in the previous section. Phylogenetic distance between languages was modeled assuming Brownian diffusion of traits on a single Maximum Clade Credibility language tree describing language geneology across the globe (Bouckaert et al., 2022). Geospatial proximity was calculated using geosphere (Hijmans, 2024) on geospatial data present in the *Glottolog* (Hammarström et al., 2023) for each language, assuming covariance is exponentially proportional to geographic distance.

We ran the model on the deeply sampled subset of the corpus, that is, on songs from the Indo-European (*n* = 297), Austronesian (*n* = 154), and Atlantic Congo (*n* = 92) language families (total *N* = 543). These families all diversified over the last few thousand years and together now represent ∼40% of the world’s languages^2^. Focusing on these three large families allows inferences about the relative importance of geographic proximity of other cultures and shared linguistic ancestry as factors in shaping song diversity over this period.

First, we tested the relative influence of behavioral context and language, leaving aside phylogenetic or geographic proximity. For each acoustic feature, we ran three models: a model that included a term for songs’ behavioral contexts; a model that included a term for songs’ culture via its glottocode; and a model that included both. All models show $\hat{R}$ < 1.05, suggesting convergence in sampling.

**Figure 5.**
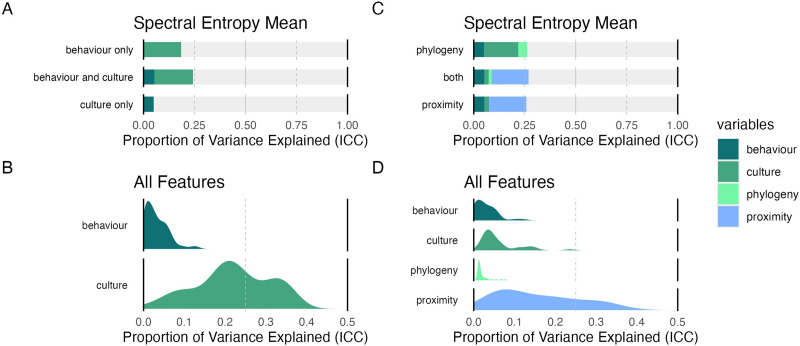
**Effects of behavioral context, culture, phylogeny and proximity on acoustic features in song**. We fit Bayesian group effects models calculating explained variance (measured by intra-class correlation, ICC) for each acoustic feature extracted from songs in the corpus. (A) We compare the ICC of models fit with behavior (coded by song type), culture (coded by language), or both for the mean spectral entropy of each song (this acoustic feature is used as an example, as in Figure 4). We note that the proportion of explained variance for behavior and culture are independent for this and other acoustic features (not shown), such that including both factors additively increases the total explained variance. (B) The distribution of ICC of behavior and culture from the model which includes both over all 38 acoustic features. Variance captured by behavioral context tends to be consistent but lower across the acoustic features, whereas variance captured by culture tends to be more variable. (C) We compared models for spectral entropy which additionally included phylogenetic distance and/or geographical distance, which we can take as proxies for vertical and horizontal transmission, respectively. We see that the explained variance of culture is largely absorbed by geographical proximity, while variance explained by phylogeny is relatively low. Additionally, there is little additional explained variance gained from including these two new factors, a trend which holds for all acoustic features. (D) We show the ICC across all acoustic features from models which include all four factors. We see that the explained variance of proximity has absorbed much of the ICC previously attributed to culture, while ICC for phylogeny is consistently quite low.

Figure 5A shows an example of ICCs across these three models for a single feature (here again, for illustration, we use *spectral entropy*). The explained variance from behavioral context and culture are independent, evidently, in that the total explained variance for the model that includes both is roughly the sum of the models that include the terms individually. This demonstrates that *both* behavioral context and culture are important factors shaping the variability in the sounds of songs.

This pattern repeated across other measured acoustic features: density plots of the ICC across all acoustic features for the models that include both behavioral context and culture show effects of both behavioral context and culture, but in general, culture tends to have a larger proportion of explained variance (Figure 5B). That is, songs are more similar in their acoustic features to each other when grouped by language than by behavior.^3^

Last, we repeated these analyses, adding terms to the model that compare the effects of phylogeny and geographical proximity, to ask whether similarities between acoustic features are better explained by horizontal transmission (transfer of features between cultures in close geographic proximity) or vertical transmission (transfer of features through descent groups within a lineage), independently of culture or behavioral context. The phylogenetic proximity was calculated as covariance matrices derived from the global tree (Bouckaert et al., 2022) while geographical proximity used coordinates associated with glottocodes (Hammarström et al., 2023).

As in the first analysis, our strategy was to model the two features of interest separately and then together in a third model. Figure 5C shows the resulting ICCs for spectral entropy; geographical proximity absorbs much of the explained variance originally attributed to culture, while phylogeny explains a much smaller proportion. This trend apparently generalizes to other acoustic features, as in Figure 5D, which shows the distributions of ICCs from models that include both phylogeny and proximity for all acoustic features.

The ICCs for phylogenetic proximity are low, in general, suggesting that horizontal transmission across language genealogies plays a more significant role in shaping the acoustics of music than does vertical transmission. Geographical and phylogenetic proximity also explained minimal variance beyond behavioral context and culture, although there is still substantial variability in the amount of variance explained across different acoustic features.

## GENERAL DISCUSSION

We report the *Expanded Natural History of Song Discography* and describe its applications for cross-cultural research on the universality and diversity of human song, with two proofs of concept. First, LASSO classifiers showed that the behavioral contexts of songs are detectable on the basis of their acoustic features, with variability in accuracy across contexts. Second, Bayesian group-effects modeling showed that the language and behavioral context of songs explains variability in acoustics independently, with further influences of language ancestry and geographic proximity.

The *Expanded Natural History of Song Discography* is permanently archived at https://doi.org/10.5281/zenodo.8223168 and we encourage members of the research community to use this resource in their research.

Many questions should be testable with this corpus. For example, in conjunction with large multi-lingual speech corpora, such as *Common Voice* (Ardila et al., 2020) and *DoReCo* (Seifart et al., 2024), one might study how speech and song vocalizations may co-vary across cultures and contexts. Research applying the audio files in citizen-science experiments might investigate how the original behavioral context may shape or constrain emotional reactions to the music, allowing us to better understand the evolutionary underpinnings of musical behaviors. Studies on psychological responses to music may sample stimuli from the corpus, as in previous work (Bainbridge et al., 2021), so as to improve the generalizability of research which necessarily use a small number of stimuli (see Hilton & Mehr, 2022).

We note that the 10-second duration of these samples could be a limitation for some researchers. While some auditory perception studies use around stimuli of about this duration (e.g., ∼13 s in a study of neural entrainment to rhythm; Doelling & Poeppel, 2015) or shorter (e.g., 3.7 s in Merrill et al., 2012), some studies of tapping to a musical beat use longer stimuli (e.g., 20–30 seconds in Martens (2011) and De Bruyn et al. (2008)).

One important long-term use for corpora involves new data. We hosted the corpus on *D-PLACE* to help users easily link the songs from this corpus to cultural and ecological data from other corpora. The provision of raw audio files also means researchers can experiment with different methods of musical and acoustic feature extraction. For the purposes of this paper, we used only single-valued estimates of acoustic features automatically extracted with an off-the-shelf tool (MIRtoolbox), but this approach has many limitations (for instance, a single estimate of tempo does not account for a song speeding up or slowing down). Richer data characterizing the musical features of each song, such as changes in features over time or transcriptions created with pitch-extraction software, may support more robust tests of cultural transmission and variability in music (see Mehr et al., 2019 for discussion).

More fundamentally, the question of what to transcribe from a song, and how, is a longstanding issue in both ethnomusicology (Lomax, 1980) and music technology (Benetos et al., 2013) (for discussion, see Supplementary Text 1.2.5 in Mehr et al., 2019). In addition to the technical concern of what tools to use, the scientific question of what constitutes a meaningful acoustic feature depends on the research question at hand. For some questions, the most informative quantitative representation of musical information may be at a higher level than what MIRtoolbox is designed to extract from audio. We encourage those using the corpus to publish open-access data representing the musical and acoustic information it contains, so as to increase its utility for the community.

We anticipate that future iterations of this corpus would also benefit from expanded metadata and annotations. For example, reporting the degree of confidence alongside each coding decision has been argued to improve transparency when reporting cross-cultural databases, similar to how it is standard practice to report measures of inter-rater reliability when multiple researchers ascribe codes to the same data (Slingerland et al., 2020). Indeed, some songs could plausibly be described as having more than one behavioral context, but such information is not captured by the current version of the corpus. A potential solution is to include a secondary behavioral context when applicable or refine our behavioral taxonomy as we learn more about form-function relationships in music. The inclusion of multiple labels in this fashion may aid the evaluation of classification accuracy (McKay & Fujinaga, 2006).

Relatedly, some basic information is not yet available for the song excerpts in the corpus. The age of each recording is not yet catalogued; this type of data is, perhaps surprisingly, often difficult to reliably annotate. In the first iteration of the Natural History of Song Discography (Mehr et al., 2019), recording year is only haphazardly reported, and where information is present it varies widely in specificity (e.g., “1966”; “1923–1933”; “pre-2011”). Those interested in archival work may contribute to the corpus by systematically expanding its annotations.

Lastly, we encourage researchers to test hypotheses about the evolution of music using this corpus, as well as to connect it to other datasets that reveal how song evolution may be driven by cultural and ecological factors, such as the diffusion of musical instruments. Such questions have a long history in computational analyses of language, with phylogenetic analyses of this kind revealing that languages evolve in rapid bursts (Atkinson et al., 2008); a mapping of the expansion of Austronesian (Gray et al., 2009), Indo-European (Bouckaert et al., 2012), Pama-Nyungan (Bouckaert et al., 2018), and Sino-Tibetan (Sagart et al., 2019; Zhang et al., 2019) language families; and the finding that word order can be explained as a function of linguistic lineage rather than Chomskyan universality (Dunn et al., 2011). As the study of the evolutionary history of human vocalization grows, we hope that this corpus will provide a systematically constructed foundation for those curious about how musicality is instantiated worldwide.

## ACKNOWLEDGMENTS

We thank Micah Walter, Brisa Garcia, and Nivi Ravi for contributions to searches for candidate items; Sumi Onoe for contributions to corpus validation; Luke Glowacki for contributing key ideas and support early in the project; and the members of The Music Lab for feedback and discussion.

## FUNDING INFORMATION

This research was supported by the US National Institutes of Health Director’s Early Independence Award DP5OD024566 (S.A.M.); the Royal Society of New Zealand Te Apārangi Rutherford Discovery Fellowship RDF-UOA2103 (S.A.M. and M. Snarskis); the Marsden Fund Standard Grant MFP-UOA2133 (S.A.M. and M. Snarskis); the Royal Society of New Zealand Marsden Standard Grant 20-UOA123 (Q.D.A.); the Fonds de Recherche du Québec Nature et Technologies PR-299652, to Sarah C. Woolley (supporting M.B.); and a doctoral training scholarship from Fonds de Recherche du Québec Nature et Technologies (M.B.).

## AUTHOR CONTRIBUTIONS

S.A.M. and M. Singh conceived of the research, with design contributions from Q.D.A. and T.K., S.A.M. and Q.D.A. provided funding. The initial search for candidate songs for the corpus was conducted by A.B., D.S., H.D., L.D., C.M.B., N.M., T.I., K.L., J.K., and J.L., under the supervision of S.A.M., S.A., and M.B. The process for sourcing audio recordings was developed and supervised by L.Y. and C.M.B., and items were sourced by L.Y., C.M.B., J.S.L., and M.B. The songs were validated by M.B., C.B.H., A.K., T.K., J.S.L., A.M., V.M., M. Shin, and M. Snarskis. M. Snarskis and M.B. conducted analyses and created visualizations, with contributions from S.A.M. M.B., M. Snarskis, and S.A.M. led the writing of the manuscript, and all authors approved it.

## DATA, CODE, AND MATERIALS AVAILABILITY

The Expanded Natural History of Song Discography is freely available at https://doi.org/10.5281/zenodo.8223168. A fully reproducible manuscript; data; analysis and visualization code; and other materials are available at https://github.com/themusiclab/nhs-expanded; this repository is permanently archived on Zenodo at https://doi.org/10.5281/zenodo.15717354.

## Notes

^1^ With the exception of one North American subregion, “Regional, Ethnic, and Diaspora Cultures”, which had ambiguous boundaries.^2^ At time of writing, Glottocode documents 3270 child languages of these three language families, out of 8605 total languages documented.^3^ Note, however, that behavior is limited to 10 different factors, one for each context, while language uses 188 different factors. A finer- or coarser-grained taxonomy of behavioral contexts could change this result.
